# Patient and health system factors associated with pretreatment loss to follow up among patients diagnosed with tuberculosis using Xpert® MTB/RIF testing in Uganda

**DOI:** 10.1186/s12889-020-09955-0

**Published:** 2020-12-03

**Authors:** Stella Zawedde-Muyanja, Achilles Katamba, Adithya Cattamanchi, Barbara Castelnuovo, Yukari C. Manabe

**Affiliations:** 1 The Infectious Diseases Institute, College of Health Sciences, Makerere University, P.O. Box 22418, Kampala, Uganda; 2 Department of Medicine, School of Medicine, Makerere University College of Health Sciences, Kampala, Uganda; 3 Division of Pulmonary and Critical Care Medicine and Center for Tuberculosis, University of California San Francisco, San Francisco, USA; 4 Division of Infectious Diseases, Department of Medicine, Johns Hopkins University School of Medicine, Baltimore, MD USA

**Keywords:** Pretreatment loss to follow-up, Xpert testing, Tuberculosis, Uganda

## Abstract

**Background:**

In 2018, Uganda started only 65% of persons with incident tuberculosis on treatment. Pretreatment loss to follow up is an important contributor to suboptimal treatment coverage. We aimed to describe the patient and health facility-level characteristics associated with pretreatment loss to follow up among patients diagnosed with pulmonary tuberculosis at public health facilities in Uganda.

**Methods:**

At ten public health facilities, laboratory register data was used to identify patients aged ≥ 15 years who had a positive Xpert®MTB/RIF test. Initiation on TB treatment was ascertained using the clinical register. Factors associated with not being initiated on TB treatment within two weeks of diagnosis were examined using a multilevel logistic regression model accounting for clustering by health facility.

**Results:**

From January to June 2018, 510 patients (61.2% male and 31.5% HIV co-infected) were diagnosed with tuberculosis. One hundred (19.6%) were not initiated on TB treatment within 2 weeks of diagnosis. Not having a phone number recorded in the clinic registers (aOR 7.93, 95%CI 3.93–13.05); being HIV-infected (aOR 1.83; 95% CI: 1.09–3.26) and receiving care from a high volume health facility performing more than 12 Xpert tests per day (aOR 4.37, 95%CI 1.69–11.29) and were significantly associated with pretreatment loss to follow up.

**Conclusion:**

In public health facilities in Uganda, we found a high rate of pretreatment loss to follow up especially among TBHIV co-infected patients diagnosed at high volume health facilities. Interventions to improve the efficiency of Xpert® MTB/RIF testing, including monitoring of the TB care cascade should be developed and implemented.

**Supplementary Information:**

The online version contains supplementary material available at 10.1186/s12889-020-09955-0.

## Background

Uganda is one of the 30 high tuberculosis(TB) and HIV burden countries [1]. In 2015, the estimated TB incidence was 253/100,000 population, a rate higher than previously projected [2]. The end TB strategy mandates a 90% reduction in TB incidence (compared to 2015) by 2035 [3]. In order to achieve this target, high burden countries including Uganda must break the transmission cycle by diagnosing and placing on appropriate therapy, at least 90% of all persons with TB annually [3]. However, TB treatment coverage (the ratio of notified to estimated persons with TB) in Uganda has been persistently low and in 2018, only 65% of all estimated persons with TB were started on treatment by the National TB and Leprosy Program (NTLP) [1].

Persistently low TB treatment coverage is a result of a leaky “cascade of care”. The “cascade of care” is a series of sequential steps that patients must successfully complete in order to achieve a desired outcome (cure or control) for a disease of interest [4]. The TB cascade of care, derived from the World Health Organization (WHO) Onion model [5] outlines the implementation steps for patients to achieve TB cure: they must recognize TB signs and symptoms; present to health facilities; be recognized by the healthcare system; receive a microbiological test for TB; be started on TB treatment and be retained in care for the entire duration of treatment. Previous studies have shown that a significant proportion of TB patients do not recognize the signs and symptoms of TB and therefore do not present to the healthcare system [2, 6]. Of those who present, only 20% of receive a microbiological test for TB [2, 7, 8]. Of those tested and diagnosed with TB, 20–25% never start treatment as a result of pretreatment loss to follow-up(LFU) [4, 7, 9].

Pretreatment LFU is defined as the loss of patients between diagnosis with TB and treatment initiation and is a critical point of attrition in the “cascade of care”. Patients who are lost to follow-up before starting TB therapy continue to transmit within communities and have significantly worse disease outcomes including death [7]. In other settings, pretreatment LFU has been associated with older age (> 45 years), male sex, and receiving care from high volume tertiary hospitals [10–12]. We sought to describe patient and health facility level characteristics associated with pretreatment LFU up among patients with pulmonary bacteriologically confirmed (PBC) TB using Xpert® MTB/RIF testing at public health facilities in Uganda.

## Methods

### Study setting

Healthcare delivery in Uganda is tiered with primary, secondary and tertiary levels of care. Within the healthcare system, TB care services are provided at all secondary and tertiary levels of care, as well as selected primary care facilities. Over the past five years, sputum microscopy, previously the main diagnostic test for TB, has been increasingly replaced by Xpert®MTB/RIF testing. To date, about 235 health facilities (15% of all health facilities which offer TB care services) are equipped with Xpert® MTB/RIF machines. At these health facilities, Xpert®MTB/RIF testing is the initial diagnostic test for all patients with signs and symptoms of TB [13]. Health facilities which do not have Xpert®MTB/RIF machines use sputum microscopy as the mainstay of diagnosis, but access Xpert® MTB/RIF testing for selected patient populations (e.g., patients infected with HIV and those previously treated for TB) through a specimen referral system. Health facilities with Xpert®MTB/RIF testing act as “diagnostic hubs” for lower health facilities within a 20–30 km radius. Sputum samples are transported by motorcycle to the diagnostic hubs and results are returned to the referring health facilities by the same courier. All patients diagnosed with TB are recorded in standardized paper-based national register.

Unless there is an indication for hospital admission, TB treatment is offered free-of-charge in the outpatient setting. The Uganda National TB and Leprosy program (NTLP) recommends that all patients diagnosed with drug-susceptible TB are started on treatment as soon as possible, preferably within 24 h [13]. For patients who prefer to complete TB treatment at a health facility other than the one where TB diagnosis is made, the NTLP still recommends that TB treatment is started at the diagnosing facility and the patient is subsequently referred to the health facility of their choice.

The NTLP uses separate national, standardized, paper-based registers for recording and reporting: all patients with signs and symptoms of TB (TB presumptive registers); all patients with a bacteriological confirmation of TB (TB laboratory registers); and all patients started on TB treatment (TB treatment registers). These registers are present at all health facilities where TB services are offered. Data for all patients started on TB treatment within each district is collated into one district TB register periodically to allow for recording of treatment outcome data for patients who may have started or completed TB treatment at a different health facility than the one where they were diagnosed.

To get a representative picture of the healthcare system, we purposively selected for this study, health facilities from different levels of the healthcare system (three primary care facilities, four district hospitals and three tertiary referral hospitals) across ten districts in Uganda. All facilities in this study have Xpert® MTB/RIF testing available onsite and use this test as the initial diagnostic test for patients presenting with signs and symptoms of TB [13]. All facilities in this study also act as diagnostic hubs and receive additional samples for testing from primary care facilities within their catchment area.

### Data collection

We carried out a retrospective review of data collected at the selected health facilities between January 1st and June 30th, 2018. We used data from laboratory registers to identify patients aged ≥ 15 years who had a positive Xpert®MTB/RIF test and were rifampicin sensitive. We excluded all patients who had an Xpert®MTB/RIF test done as part of treatment monitoring. We then compared diagnostic data with treatment initiation records in the health facility TB treatment registers and in the district TB registers (for patients who could have started on TB treatment at a different health facility within the same district). Patients were considered to have experienced pretreatment LFU if their names were in the laboratory register but no evidence of their names in the health facility clinic or district TB registers within 2 weeks of diagnosis.

Data on patient characteristics including age, sex, HIV status, ART status, and residence was collected from the laboratory registers. Patient’s age was categorized in 10-year age groups starting at 15 years in according to the practices on case notifications at the district and national level. Distance to the health facility was calculated as the linear distance, based on global positioning system coordinates (QGIS Desktop, Versions 2.12.0), from the patient’s recorded residence to the health facility.

Health facility records were used to obtain data on health facility characteristics including the number of Xpert®MTB/RIF tests done, cartridge and medicine stock outs. Data on cartridge malfunction was collected by interviewing laboratory healthcare workers at each health facility.

### Data analysis

Baseline characteristics of the study population were described using frequencies and percentage. The proportion of patients who experienced pretreatment LFU was also described. A multilevel logistic regression model accounting for clustering by health facility was used to examine factors associated with pretreatment loss to follow up. A sensitivity analysis was performed using multiple imputation to examine the effect of missing data on our measures of association. For variables with missing data (distance from health facility, HIV status and ART status), we assumed data were missing at random and performed multivariate normal imputation [14] using age, sex and level of health facility as predictor variables. All data analyses were carried out using STATA® version 13.

## Results

**Fig. 1:**
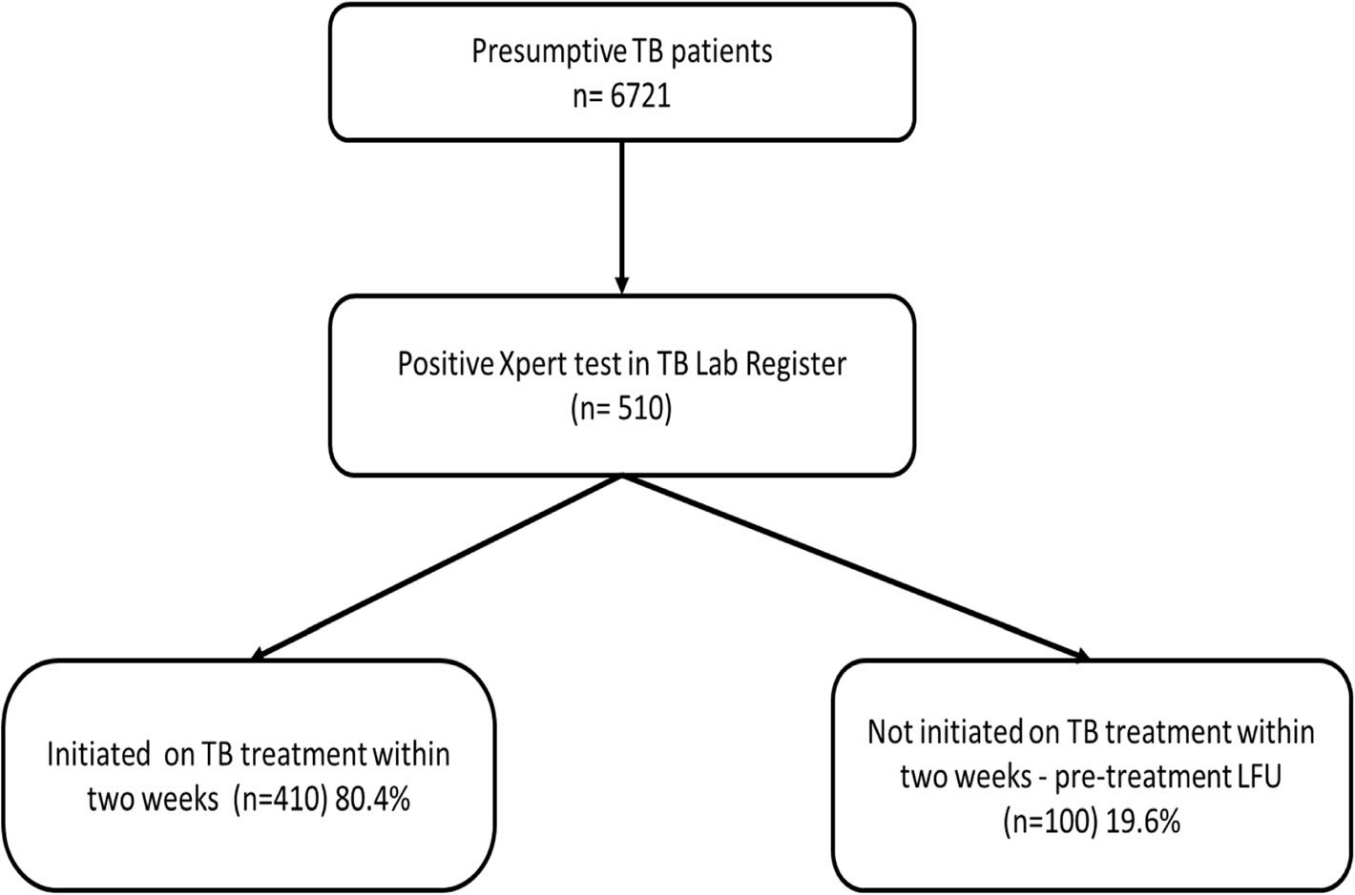
Patient Flow Chart

From January to June 2018, 6721 persons with presumptive TB were tested with Xpert®MTB/RIF at the ten health facilities. Of these, 510 (7.6%) tested positive for MTB and 410 (80.4%) were started on TB treatment. (Fig. 1).

Table 1 shows the characteristics of study participants. Patients enrolled in the study were predominantly male (61.2%). The majority (*N* = 244, 47.9%) were diagnosed at tertiary referral hospitals. Only a third of all patients were started on TB treatment on the same day. HIV status was available for 479 (94.0%) study participants and 161 (31.5%) of these were HIV co-infected. ART status was available for 138 (85.7%) HIV co-infected patients. Among these, 101 (73.2%) were on ART prior to starting TB treatment while 37 (26.8%) were newly diagnosed with HIV and initiated ART after starting TB treatment (Table 2).

**Table 1:** Characteristics of Study Participants

Individual Level Characteristics (***N*** = 510)	
Characteristic	N (%)
**Sex**	
Male	312 (61.2)
Female	198 (38.8)
**Age**	
15–24	115 (22.7)
25–34	162 (31.7)
35–44	111 (21.7)
45–54	66 (12.9)
> 55	56 (10.9)
**Phone No.**	
Yes	323 (63.3)
No	187 (36.7)
**Distance from health facility (*n*** **= 469)**	
< =5 km	132 (28.1)
6-20 km	122 (26.0)
21-35 km	142 (30.2)
> 35 km	73 (15.6)
**HIV status (*n*** **= 479)**	
HIV positive	161 (31.5)
HIV negative	318 (66.4)
**ART Status (*n*** **= 138)**	
On ART before TB diagnosis	101 (73.2)
Started ART after TB diagnosis	37 (26.8)
**Facility Level Characteristics**	
**Characteristic**	N(%)
**Health Facility Level**	
Primary care Facility	122 (23.9)
District Hospital	144 (28.2)
Tertiary Hospital	244 (47.9)
**Number of Xpert tests done**	
< =8 tests/day	342 (67.1)
9–12 tests/day	89 (17.4)
> 12 tests/day	79 (15.5)
**Xpert Turnaround time**	
< 24 h	173 (34.0)
24–48 h	164 (32.0)
> 48 h	173 (34.0)
**Xpert Module Malfunction (past 3 months)**	
No	356 (69.8)
Yes	154 (30.2)
**Cartridge Stock Outs (past 3 months)**	
Yes	158 (30.9)
No	352 (69.1)
**Medicine Stock out (past 3 months)**	
Yes	67 (13.1)
No	443 (86.9)

**Table 2:** Patient level factors associated with pretreatment loss to follow up in a multilevel logistic regression model

Characteristic	Initiated on Rx *N* = 410	Not Initiated on Rx *N* = 100	Crude Odds Ratio (95% CI)	Adjusted Odds Ratio (95% CI)
Sex				
Male	256 (82.1)	57 (17.9)	reference	–
Female	154 (77.8)	43 (22.2)	1.30 (0.84–2.04)	–
Age				
15–24	92 (80.0)	23 (20.0)	reference	–
25–34	130 (80.3)	32 (19.7)	0.98 (0.54–1.79)	–
35–44	91 (81.9)	20 (18.1)	0.88 (0.45–1.70)	–
45–54	51 (77.2)	15 (22.8)	1.17 (0.56–2.46)	–
> 55	46 (82.1)	10 (17.9)	0.88 (0.39–2.00)	–
Phone No.				
Yes	298 (92.3)	25 (8.7)	reference	reference
No	112 (59.9)	75 (40.1)	**8.99 (5.17–15.64)**	**7.93 (3.93–13.05)**
^a^Distance from health facility (*n* = 469)				
> 35 km	64 (87.7)	9 (12.3)	reference	–
21-35 km	124 (87.3)	18 (12.7)	1.04 (0.44–2.49)	–
6-20 km	106 (86.9)	16 (13.1)	1.12 (0.47–2.69)	–
< =5 km	103 (81.9)	29 (18.1)	2.08 (0.93–4.67)	–
^b^HIV status (*n* = 479)				
HIV negative	281 (88.4)	37 (11.6)	reference	reference
HIV positive	129 (80.1)	32 (19.9)	**1.86 (1.10–3.12**)	**1.88 (1.09–3.26)**
^c^ART Status (*n* = 138)				
On ART before TB diagnosis	91 (90.1)	10 (9.9)	reference	–
Not on ART before TB diagnosis	34 (91.9)	3 (8.1)	0.61 (0.14–2.61)	–

^a^ *41 patients (13 who were and 28 who were not initiated on treatment) did not have data on distance from health facility*

^b^*31 patients who were not initiated on TB treatment did not have HIV status recorded*

^c^*23 patients (4 who were and 19 who were not initiated on TB treatment) did not have data on ART status*

Overall, 100 (19.6%) patients were not initiated on TB treatment within 2 weeks of diagnosis (Fig. 1). On bivariate analysis, patient-level factors associated with pretreatment LFU included not having a phone number listed in the TB clinic register (OR 8.99, 95%CI 5.17–15.64) and being HIV-infected (OR 1.86; 95% CI: 1.10–3.12) (Table 2). Facility-level factors (Table 3) associated with pretreatment LFU included being diagnosed at a health facility performing > 12 Xpert tests per day (OR 2.30, 95%CI 1.77–2.99); being diagnosed at a health facility with Xpert cartridge stock outs in the past 3 months (OR 1.63; 95% CI: 1.04–2.54); and being diagnosed at a health facility which experienced module malfunction in the past 3 months (OR 2.11; 95% CI: 2.53–2.89).

**Table 3:** Health facility factors associated with pretreatment loss to follow up in a multilevel logistic regression model.

Characteristic	Initiated on Rx ***N*** = 410	Not Initiated on Rx ***N*** = 100	Crude Odds Ratio (95% CI)	Adjusted Odds Ratio (95% CI)
**Number of Xpert tests done**				
< =8 tests/day	280 (81.9)	62 (18.1)	reference	reference
9–12 tests/day	77 (86.5)	12 (13.5)	0.84 (0.69–1.00)	0.53 (0.24–1.17)
> 12 tests/day	53 (67.1)	26 (32.9)	**2.30 (1.77–2.99)**	**4.37 (1.69–11.29)**
**Xpert module malfunction (past 3 months)**				
No	295 (82.9)	61 (17.1)	reference	reference
Yes	115 (74.7)	39 (25.3)	**1.63 (1.04–2.54)**	1.57 (0.86–2.85)
**Cartridge stock outs (past 3 months)**				
No	115 (72.8)	43 (27.2)	reference	reference
Yes	295 (83.8)	57 (16.2)	**2.11 (1.53–2.89)**	1.59 (0.88–2.86)
**Medicine stock out (past 3 months)**				
No	357 (80.6)	86 (19.4)	reference	–
Yes	53 (79.1)	14 (20.9)	1.11 (0.56–2.18)	–

In the adjusted analysis (Tables 2 and 3), only three factors – not having a phone number listed in the TB clinic register (aOR 7.93, 95%CI 3.93–13.05); being HIV-infected (aOR 1.83; 95% CI: 1.09–3.26) and being diagnosed at a health facility performing more than 12 Xpert tests per day (aOR 4.37, 95%CI 1.69–11.29) remained significantly associated with pretreatment LFU. In sensitivity analyses using multiple imputation, all significant associations were maintained (Supplementary Tables 1 & 2).

## Discussion

In this retrospective study we examined patient and health facility factors associated with pretreatment LFU at public health facilities in Uganda; we found that about one in five patients diagnosed with TB experienced pretreatment LFU. Pretreatment LFU is a persistent problem in public health systems high TB burden settings [15–17]. In India, one of the countries with the highest TB burden in the world, pretreatment LFU is estimated to be responsible for at least 8% (200,000) of all missing persons with TB annually [4]. In our study, the observed proportion of patients experiencing pretreatment LFU would translate into 11% (10,000) of all missing persons with TB countrywide in that period.

Earlier studies examining pretreatment LFU among patients diagnosed with sputum microscopy showed that increased time and monetary costs associated with returning to health facilities to deliver a second sputum sample and/or collect sputum results were partly responsible for observed high rates of pretreatment LFU [8, 18, 19]. Xpert® MTB/RIF testing, a near POC test that requires only one sputum sample and has relatively quick turnaround times held the promise of reducing pretreatment LFU. Results from one clinical trial conducted in South Africa showed a reduction in pretreatment LFU driven by the increased proportion of patients who received a same-day diagnosis [20]. However, this finding has not been replicated in routine care settings both in South Africa and Uganda [16, 21, 22]. Patients accessing Xpert®MTB/RIF testing in our setting still experience relatively long turnaround times [21]. In our study, only one-third of patients received a same-day diagnosis.

High patient volumes (measured in our study by the number of Xpert® MTB/RIF tests run each day) likely prolong the turnaround time for Xpert® MTB/RIF testing, and result in more patients experiencing pretreatment LFU. This association between high patient volumes and pretreatment LFU has been shown in Asia and other parts of sub-Saharan Africa [10, 11, 23, 24] and has been attributed to prolonged clinic waiting times, and increased laboratory turnaround times for sputum microscopy. In the Ugandan setting, high patient loads also make it harder to monitor treatment initiation among patients diagnosed with TB. The current system to monitor treatment initiation requires healthcare workers to manually reconcile laboratory registers with treatment registers, a task that may be difficult to perform regularly at health facilities with high patient volumes. At these health facilities, electronic systems that carry out real time monitoring of patient retention along the cascade of care could lead to reductions in pretreatment LFU. Although these kinds of electronic data innovations are commonplace in HIV care, they remain largely unutilized for TB care [25].

HIV-infected patients had higher rates of pretreatment LFU in our study consistent with data from other high TBHIV burden settings [17, 26]. Late presentation to care could partially account for these LFU patients. In Malawi, advanced HIV disease was shown to result in suboptimal linkage to TB treatment as patients were often too sick to return to the health facility for their results or died before treatment initiation [18]. In Zimbabwe, close to 50% of pretreatment LFU was due to deaths before treatment initiation particularly among HIV-infected patients [17]. In our study, late presentation to care was examined by analyzing the ART status of patients who were started on TB treatment. Among those patients whose ART status was available, about a quarter (27%) initiated ART after starting TB treatment. This is consistent with routine surveillance data from the AIDS Control Program that shows that despite the roll out of “test and treat” for HIV, about 30% of all newly diagnosed HIV patients still present with Stage III and IV disease [27]. The introduction of additional point-of-care tests with shorter turnaround times e.g. lateral flow urine lipoarabinomannan (LF-LAM) [28] into the diagnostic algorithm for patients with Stage III and IV disease may improve linkage to treatment among this group of patients.

In our study, patients who did not have a phone number recorded was strongly associated with pretreatment LFU. Although patients may deliberately decline to divulge their phone numbers due to self-stigma related to TB [29], the proportion of patients with a recorded phone number in our study (63%) was comparable to the national phone coverage for rural areas (65.7%) [30] and is therefore likely to represent actual phone ownership. Patients without phone numbers may belong to a lower socio-economic class and may lack the financial means to return to health facilities to receive their results and start on TB treatment [31]. The recently concluded patients’ costs survey in Uganda showed half of all TB patients incurred catastrophic TB care costs which were mainly driven by nonmedical expenditure such as travel [32]. Interventions to reduce these costs for the most vulnerable patients e.g. prioritizing them for same-day diagnosis or provision of socioeconomic support may reduce pretreatment LFU. Similarly, community tracing interventions, where community healthcare workers conduct home visits to trace patients with no phones would also help reduce pretreatment LFU.

Consistent with studies from Ghana [10] and other settings in Uganda [33], there was no association between distance from the health facility and pretreatment LFU. This may be due to the decentralized nature of health service delivery in Uganda where patients access care at health facilities closest to their homes. In our study, nearly half of all patients resided within 20kms of the health facility.

### Study strengths and limitations

Our study used data collected from different levels of the healthcare system. It is therefore likely that these findings are representative of and generalizable to the public healthcare system in Uganda. However, because data for this study was collected under routine programmatic conditions, missing data may have introduced bias into our study resulting in an overestimation of pretreatment LFU. This was minimized by triangulating many data sources within the healthcare facilities and at the district level.

## Conclusion

In public health facilities in Uganda, we found a high rate of pretreatment LFU. Interventions to improve the efficiency of Xpert® MTB/RIF testing should be developed and implemented. These efforts should be targeted at large volume tertiary hospitals and at patient groups at the highest risk of pretreatment loss to follow-up.

## Supplementary Information

**Additional file 1: Supplementary Table 1**: Patient level factors associated with pretreatment loss to follow-up in a multilevel logistic regression model after multiple imputation**.**

**Additional file 2: Supplementary Table 2**: Health facility level factors associated with pretreatment loss to follow-up in a multilevel logistic regression model after multiple imputation.

## Data Availability

The datasets used and/or analyzed during the current study are available from the corresponding author on reasonable request.

## Abbreviations

aOR: Adjusted odds ratio
CI: Confidence interval
HIV: Human immunodeficiency syndrome
KMS: Kilometers
LF-LAM: Lateral flow urine lipoarabinomannan
LFU: Loss to follow-up
NTLP: National TB and Leprosy program
OR: Odds ratio
TB: Tuberculosis
WHO: World Health Organization
