# Supplementary material for: Patient and health system factors associated with pretreatment loss to follow up among patients diagnosed with tuberculosis using Xpert® MTB/RIF testing in Uganda
Source: BMC Public Health. 2020 Dec 3;20:1855. doi: 10.1186/s12889-020-09955-0 (PMC7713043; doi:10.1186/s12889-020-09955-0)
Supplement: Supplementary file 1 — Additional file 1: Supplementary Table 1: Patient level factors associated with pretreatment loss to follow-up in a multilevel logistic regression model after multiple imputation. [file 12889_2020_9955_MOESM1_ESM.docx]

**Supplementary Table 1: Patient level factors associated with pretreatment loss to follow-up in a multilevel logistic regression model after multiple imputation**

| Characteristic | Initiated on Rx  N= 410 | Not Initiated on Rx  N = 100 | Crude Odds Ratio  (95% CI) | Adjusted Odds Ratio  (95% CI) |
| --- | --- | --- | --- | --- |
| Sex | | | | |
| Male | 256 (81.5) | 58 (18.5) | reference | - |
| Female | 154 (78.6) | 42 (21.4) | 1.30 (0.84-2.04) | - |
| Age | | | | |
| 15-24 | 92 (80.0) | 23 (20.0) | reference | - |
| 25-34 | 130 (80.3) | 32 (19.7) | 0.98 (0.54-1.79) | - |
| 35-44 | 91 (81.9) | 20 (18.1) | 0.88 (0.45-1.70) | - |
| 45-54 | 51 (77.2) | 15 (22.8) | 1.17 (0.56-2.46) | - |
| >55 | 46 (82.1) | 10 (17.9) | 0.88 (0.39-2.00) | - |
| Phone No. | | | | |
| Yes | 298 (92.3) | 25 (8.7) | reference | reference |
| No | 112 (59.9) | 75(40.1) | **8.99 (5.17-15.64)** | **14.47 (6.94- 22.42)** |
| Distance from health facility (n=469) | | | | |
| >35 km | 64 (87.7) | 9 (12.3) | reference | - |
| 21-35km | 124 (87.3) | 18 (12.7) | 1.04 (0.44 -2.49) | - |
| 6-20km | 106 (86.9) | 16 (13.1) | 1.12 (0.47 -2.69) | - |
| <=5km | 103 (81.9) | 29 (18.1) | 2.08 (0.93 - 4.67) | - |
| HIV status (n=479) | | | | |
| HIV negative | 281 (88.4) | 37 (11.6) | reference | reference |
| HIV positive | 129 (80.1) | 32 (19.9) | **2.08 (1.23-3.34)** | **2.05 (1.16 -3.60)** |
| ART Status (n=138) | | | | |
| On ART before TB diagnosis | 91 (90.1) | 10 (9.9) | reference | - |
| Not on ART before TB diagnosis | 34 (91.2) | 3 (8.1) | 0.61 (0.14 -2.61) | - |

*‡ 41 patients (13 who were and 28 who were not initiated on treatment) did not have data on distance from health facility ₽31 patients who were not initiated on TB treatment did not have HIV status recorded *23 patients ( 4 who were and 19 who were not initiated on TB treatment) did not have data on ART status*
