# Supplementary material for: Patient and health system factors associated with pretreatment loss to follow up among patients diagnosed with tuberculosis using Xpert® MTB/RIF testing in Uganda
Source: BMC Public Health. 2020 Dec 3;20:1855. doi: 10.1186/s12889-020-09955-0 (PMC7713043; doi:10.1186/s12889-020-09955-0)
Supplement: Supplementary file 2 — Additional file 2: Supplementary Table 2: Health facility level factors associated with pretreatment loss to follow-up in a multilevel logistic regression model after multiple imputation. [file 12889_2020_9955_MOESM2_ESM.docx]

**Supplementary Table 2: Health facility level factors associated with pretreatment loss to follow-up in a multilevel logistic regression model after multiple imputation**

| **Characteristic** | **Initiated on Rx**  **N= 410** | | **Not Initiated on Rx**  **N = 100** | **Crude Odds Ratio**  **(95% CI)** | **Adjusted Odds Ratio**  **(95% CI)** |
| --- | --- | --- | --- | --- | --- |
| **Number of Xpert tests done** | | | | | |
| <=8 tests/day | 280 (81.9) | | 62 (18.1) | reference | reference |
| 9-12 tests/day | 77 (86.5) | | 12 (13.5) | 0.84 (0.69-1.00) | 0.53 (0.24-1.17) |
| >12 tests/day | 53 (67.1) | | 26 (32.9) | **2.30 (1.77-2.99)** | **2.50 (1.47- 4.25)** |
| **Xpert module malfunction (past 3 months)** | | | | | |
| No | 295 (82.9) | | 61 (17.1) | reference | reference |
| Yes | 115 (74.7) | | 39 (25.3) | **1.63 (1.04- 2.54)** | 1.57 (0.86 – 2.85) |
| **Cartridge stock outs (past 3 months)** | | | | | |
| No | 115 (72.8) | 43 (27.2) | | reference | reference |
| Yes | 295 (83.8) | 57 (16.2) | | **2.11 (1.53-2.89)** | 1.59 (0.88- 2.86) |
| **Medicine stock out (past 3 months)** | | | | | |
| No | 357 (80.6) | | 86 (19.4) | reference | - |
| Yes | 53 (79.1) | | 14 (20.9) | 1.11 (0.56-2.18) | - |
